# Correlates of cognitive impairment in the elderly in China: A cross-sectional study

**DOI:** 10.3389/fpubh.2022.973661

**Published:** 2022-10-20

**Authors:** Yuan-yuan Wang, Min Zhang, Xiao-xian Wang, Song Liu, Hong Ding

**Affiliations:** ^1^School of Public Health and Health Management, Anhui Medical College, Hefei, China; ^2^School of Health Management, Anhui Medical University, Hefei, China

**Keywords:** cognitive impairment, the elderly, decision tree, China, CHARLS

## Abstract

**Background:**

To identify correlates of the incidence of cognitive impairment among older Chinese populations through the use of logistic regression analysis-based decision tree approaches.

**Methods:**

Correlates of cognitive impairment among older Chinese adults were identified through logistic regression analyses, with significant variables subsequently being incorporated into a decision tree analysis, with the CHAID method being employed for pre-pruning.

**Results:**

The risk score derived from the combination of logistic regression and decision tree analyses (0.237) was lower than that derived from a decision tree analysis alone (0.389). The primary factors related cognitive impairment in this patient population included age, gender, residence status, physical health status, and caring for grandchildren.

**Conclusion:**

A combination of logistic regression and decision tree analyses can lower predicted risk scores, enabling the subdivision of populations with different characteristics and providing intuitive and specific insight regarding the effects of individual variables on predictive analyses. Overall, these results suggest that older adults in rural areas of China should be the focus of further cognitive impairment screening and interventions, particularly for older women.

## Introduction

Dementia is the third-highest burden of disease after cancer and cardiovascular disease, and in 2019 (1), dementia patients aged 65 and older spent a combined $290 billion on healthcare, long-term care and hospice services (2). Early detection, early diagnosis and early treatment were currently the most cost-effective measures to control dementia (3). Cognitive impairment (CI) is an intermediate process in the transition to dementia, characterized by mild but marked deficits in memory and/or other thinking abilities (4), affecting activities of daily living, mostly in older adults.

According to data from the seventh national Chinese census, there were 264.02 million adults 60 years of age or older in China, accounting for 18.70% of the total population. A number of studies on community-based elderly screening in China show that the prevalence of cognitive impairment in the elderly ranges from 4.2 to 44.2%.^4^ One study showed that the prevalence of cognitive impairment (MCI) among the elderly in Shenyang was 30.3%, which was significantly higher than that in the 2013 survey of Japanese elderly (18.8%) and the 2016 survey of American community elderly (21.0%) (5). Compared with normal people, the annual conversion rate of patients with mild MCI to dementia is 10 times higher (6). CI in the elderly brings great pressure and burden to the elderly themselves, their families and the society. The management and intervention of the elderly before they develop dementia can effectively delay the course of dementia (7–10). There is thus an urgent need to better define the factors associated with the incidence and progression of such cognitive impairment in an effort to better prevent its onset or mitigate associated morbidity in affected individuals (11–14).

Common risk factors for CI include demographic factors such as age, gender, and education level. These studies show that the age and gender differences of cognitive impairment were obvious, the cognitive impairment of the female elderly is more serious than that of the male, and the cognitive impairment of the older elderly is more serious than that of the younger elderly (15, 16). And now there's evidence that the higher the education level or the longer duration of education they have, the lower chance of cognitive impairment happens in older adults, and vice versa (17, 18). These factors were also relevant to cognitive impairment such as behavioral and lifestyle factors such as diet and exercise. Healthy diet and regular exercise were protective factors of cognitive impairment in the elderly (19, 20). But the disease factors such as hypertension (21, 22), diabetes (23, 24), stroke (25), and obesity (26) were risk factors for cognitive impairment in the elderly. A 3-year follow-up study on socioeconomic status and cognitive impairment in Italy found that people with cognitive impairment were generally less educated, more engaged in manual labor, and less engaged in white-collar, technical or academic work (27). At the same time, a study of four provinces in China showed that there were population differences in the distribution of MCI detection rates, and the detection rate of MCI in rural populations was higher than that in urban areas (28).

Understanding the influencing factors of CI, early intervention on modifiable factors, and preventing the occurrence of the disease have an important role. At present, a certain number of investigations and studies have been carried out on the influencing factors of CI in the elderly in China, but most of them were limited to specific regions, and there are few national samples. This study used the China Health and Retirement Longitudinal Study (CHARLS), a set of high-quality microdata representing families and individuals of middle-aged and elderly people aged 45 and above in China, to analyze the influencing factors of CI in the elderly in China. At the same time, two methods of logistic regression and decision tree model were used to analyze the influencing factors of CI in the elderly in China, and the factors correlating with CI of the elderly in China was comprehensively analyzed to provide a basis for the development of CI preventive measures.

## Methods

### Study design and data selection

The present study was developed using the data derived from the 2018 CHARLS from The Center for Healthy Aging and Development of Peking University (29, 30). This survey covered 22 provinces and autonomous regions in China home to ~85% of the overall Chinese population. The CHARLS's baseline survey includes one person per household aged 45 years of age or older and their spouse, totaling 17,708 individuals, living in 10,257 households in 450 villages/urban communities (29, 30). A stratified (by per capita GDP of urban districts and rural counties) multi-stage (county/district-village/community-household) PPS random sampling strategy was adopted. These survey data were thus highly representative of the population as a whole.

We followed the research object selection strategy of CHARLS and only selected the elderly aged 60 and above at the time of the investigation as the research object. There were 11,153 participants aged 60 and above of the total 17,708 people. The personal basic information, residence type, health status, and family structure information etc. were taken as research variables. The variables consist of basic demographics and health-related information were shown in Table 1.

**Table 1:** Single-factor analysis of cognitive impairment among elderly Chinese individuals with different characteristics.

	**Total (*N* = 11,153)**	**Cognition**		**χ^2^**	***p*-value**
		**No CI (*N* = 6,059)**	**CI (*N* = 5,094)**		
**Gender**				91.448	< 0.001
Male	5422(48.6)	3197(52.8)	2225(43.7)		
Female	5731(51.4)	2862(47.2)	2869(56.3)		
**Age(years)**				468.368	< 0.001
60–69	6807(61.0)	4111(67.8)	2696(52.9)		
70–79	3219 (28.9)	1651(27.2)	1568(30.8)		
≥80	1127(10.1)	297(4.90)	830(16.3)		
**Education level**				62.648	< 0.001
Illiterate	6029(54.0)	2770(45.7)	3259( 64.0)		
Primary	2373(21.3)	1615(26.7)	758(14.9)		
Junior high school and above	2751(24.7)	1674(27.6)	1077(21.1)		
**Marital status**				196.762	< 0.001
Married	8716(78.1)	5040 (83.2)	3676(72.2)		
Not married	2437(21.9)	1019(16.8)	1418(27.8)		
**Living at home**				6.799	0.009
Yes	10906(97.8)	5945(98.1)	4961(97.4)		
No	247(2.2)	114(1.9)	133(2.6)		
**Type of residence**				116.264	< 0.001
Urban	2676(24.0)	1696(28.0)	980(19.2)		
Rural	8477(76.0)	4363(72.0)	4114 (80.8)		
**Religious belief**				0.322	0.570
Yes	1221(10.9)	654(10.8)	567(11.1)		
No	9932(89.1)	5405(89.2)	4527(88.9)		
**Caring for grandchildren**				129.601	< 0.001
Yes	2527(22.7)	1623(26.8)	904(17.7)		
No	8626(77.3)	4436(73.2)	4190(82.3)		
**Pain**				48.470	< 0.001
Never	4221(37.8)	2371(39.2)	1850(36.3)		
Occasionally	4604(41.3)	2572(42.4)	2032(39.9)		
Often	2328(20.9)	1116(18.4)	1212(23.8)		
**Financial support**				3.510	0.061
Yes	4267(38.3)	2366(39.0)	1901(37.3)		
No	6886 (61.7)	3693(61.0)	3193(62.7)		
**Live with children**				47.710	< 0.001
Never	2913(26.1)	1696(28.0)	1217(23.9)		
≤ 6 months	1089 (9.8)	651(10.7)	438(8.6)		
>6months	7151(64.1)	3712(61.3)	3439(67.5)		

### Measurement of CI

The Mini-Mental Status Examination (MMSE) questionnaire was used to gauge CI in the subjects of this study. This questionnaire consists of 30 total items pertaining to six dimensions (orientation, attention and computation, memory, language ability, executive ability, and visuospatial ability), with correct and incorrect/unknown answers, respectively being scored with a 1 and a 0. 10 questions for orientation (e.g., ‘What is the year? Season? Date? Day of the week? Month?' and ‘Where are we now: State? County? Town/city? Hospital? Floor?'), the total score of this dimension was 10 points. 2 questions for Attention and computation (e.g., ‘The examiner names three unrelated objects clearly and slowly, then asks the patient to name all three of them.' and ‘I would like you to count backward from 100 by sevens. Stop after five answers.'), the total score of this dimension was eight points. One question for memory such as “Earlier I told you the names of three things. Can you tell me what those were?,” the total score of this dimension was three points. Two for language ability (e.g., ‘Show the patient two simple objects, such as a wristwatch and a pencil, and ask the patient to name them.'), the total score of this dimension was three points. Three for executive ability (e.g., ‘Take the paper in your right hand, fold it in half, and put it on the floor.'), the total score of this dimension was five points. One for visuospatial ability (e.g., ‘Please copy this picture.'), the total score of this dimension was one point. Total possible scores of MMSE range from 0-30, with higher scores corresponding to better cognitive function. The MMSE score thresholds used to define cognitive impairment varied as a function of subject educational level as follows: illiteracy < 17, primary school < 20, junior high school and above < 24 (31–34). To facilitate a convenient decision tree analysis approach, the level of CI was treated as a binary classification variable rated in accordance with the education level-related thresholds detailed above, combining CHARLS household questionnaire and MMSE scores to stratify patients into two groups including no cognitive impairment(no CI) coded as a 0 and cognitive impairment (CI) coded as a 1.

### Measurement of other variables

The predictor variables included gender (female, male), age(60–69, 70–79,≥80 years), marital status (married, not married), education level (Junior high school and above), Living at home(Yes, No), Type of residence (Urban, Rural), Religious belief (Yes, No), Caring for grandchildren (Yes, No), Pain (Never, Occasionally, Often), Financial support (Yes, No), Live with children (Never, ≤ 6 months, >6months).

The marital status was categorized as married, not married. If the participants answered “Married and live with spouse” or “Married but don't living with spouse temporarily for reasons such as work” to “What is your marital status?,” the marital status was defined as “married.” If the answers were “Separated, don't live together as a couple anymore” or “Divorced” or “Widowed” or “Never married,” the marital status was defined as “not married.” The education level was categorized as illiterate, primary, and junior high school and above. If the participants answered “No formal education (illiterate)” or “Did not finish primary school” for “What's the highest level of education you have now?,” the education level was defined as “illiterate.” If the participants answered “Sishu/home school” or “Elementary school,” the education level was defined as “primary.” If the participants answered “Middle school” or “High school” or “Vocational school” or “2-/3-Year College/Associate degree” and above, the education level was defined as “junior high school and above.”

The variable “living at home” was referring to a new type of care model for the elderly in China. The concept of “living at home” was highly similar to the word “community care” abroad. The definition of “living at home” was to coordinate the efforts of all social parties to jointly provide satisfactory services for the elderly at home. Convenient conditions and diversified support should be provided for the elderly at home by the family members, communities, social organizations and government departments (35). The living at home was categorized as yes and no. If the participants answered “Family housing” for “What was the type of your address,” the living at home was defined as “yes.” If the participants answered “Nursing home” or “Hospital” or “Other,” the living at home was defined as “no.”

The type of residence was categorized as town and rural for the question “Was your address in the village or city/town?.” The religious belief was categorized as yes and no for the question “Do you have any religious belief? Such as Buddhism, Taosim, Christianity, etc..” The caring for grandchildren was categorized as yes and no. If the participants answered “No” or “Have no grandchild” for the question “During last year, did you/ your spouse spend time in taking care of your grandchildren?.” The caring for grandchildren was defined as “no.” The pain was categorized as never, occasionally, and often. If the participants answered “None” for the question “Are you often troubled with any body pains?,” The pain was defined as “no.” If the participants answered “A little” or “Somewhat,” the pain was defined as “occasionally.” If the participants answered “Quite a bit” or “Very,” the pain was defined as “often.” Financial support was categorized as yes and no. If the participants answered “providing living expenses, paying for water, electricity or telephone bill, paying for mortgage/rent or other forms of regular expenses, or buying food, clothes or other stuff” for “During last year, what's the amount of financial support received from your family members?,” financial support was defined as “yes.” If the participants answered “nothing received” or “have no idea,” financial support was defined as “no.” The live with children was categorized as Never, ≤ 6 months, and >6months for the question “During last year, how many mouths had [X Child Name] lived with you and your spouse?”

Of the 11,153 individuals included in this study, 5422(48.6%) were male and 5731(51.4%) were female. People aged 60-69 were 6807 (61.0%), aged 70-79 were 3219 (28.9%), aged 80 and above were 1127 (10.1%). There were 8,716 people (78.1%) and 2,437 people (21.9%), respectively lived in urban and rural areas. There were 6029 people with education level of illiterate accounting for 54.0, primary 2373 people accounting for 21.3, then junior high school and above 2,751 people accounting for 24.7%. 6,059 and 5,094 were, respectively classified as no cognitive impairment (no CI) and cognitive impairment (CI).The percentage of other variables were shown in Table 1.

### Statistical analysis

To test the impact of regional culture on cognitive impairment, the 11,153 elderly subjects included in this study were additionally stratified into urban and rural subgroups in accordance with their reported residence type to permit comparisons between these two groups.

All data were analyzed using SPSS 22.0. A logistic regression analysis was initially used to screen for factors influencing cognitive impairment rates among elderly Chinese individuals, after which a decision tree model was established based on the data derived from the 2018 CHARLS survey. Decision tree used a tree graph as an analysis tool based the principles of probability theory. Its basic principle was to use decision point to represent decision problem, and use plan branch to represent alternative plan, then use probability branch to represent possible result of plan, through the calculation and comparison of profit and loss value of each plan under various result conditions, provided decision basis for decision maker. This decision tree analysis utilized the Chi-square automatic interactive detection (CHAID) algorithm. Demographic characteristics were analyzed, and chi-squared tests were used to detect statistical significance at an α = 0.05 level.

## Results

### Univariate analyses

Chi square test was taken to each univariate analysis. There were significant differences in the incidence of cognitive impairment among older adults in urban and rural settings, and these levels also varied significantly as a function of subject age, gender, education level, whether they reported caring for grandchildren, whether they reported any physical pain or disability, and whether they reported living with their children (*P* < 0.001). There were no differences in cognitive impairment status as a function of religious belief and financial support (*P* > 0.05). For further details, see Table 1.

### Logistics regression analysis

For logistic regression analyses, cognitive impairment level was defined based upon a combination of education level and MMSE scores and treated as a binary classification variable (no CI vs. CI). To analyze the factors related to cognitive impairment, chi square test was used to each univariate analysis. The nine variables in significant differences (except religious belief, financial) in the univariate analysis were used as the independent variables of the model for multiple logistic regression analysis. A logistic regression was subsequently used to identify factors associated with the incidence of cognitive impairment. Among the 9 variables included, there were no significant differences in the incidence of cognitive impairment among older adults in whether they reported “Living at home”(*P* > 0.05). Compared with the elderly without pain, there was no significant differences in cognitive impairment among the elderly with “Pain Occasionally” (*P* > 0.05), but there was significant differences among the elderly with “Often pain” (*P* < 0.05). For the elderly who never lived with their children, there was no significant differences between who lived with children for ≤ 6 months (*P* > 0.05), but there was significant differences between the elderly who lived for more than 6 months (*P* < 0.05), as shown in Table 2.

**Table 2:** Logistic regression analysis of factors correlating with cognitive impairment among elderly Chinese individuals with different characteristics.

	**β**	**S.E**.	**Walsχ^2^**	***p*-value**	**OR(95%CI)**
**Gender (X1)**					
Male					1.000
Female	0.365	0.038	91.187	< 0.001	1.440(1.336, 1.552)
**Age (X2)**					
60–69					1.000
70–79	0.370	0.043	73.823	< 0.001	1.448(1.331, 1.576)
≥80	1.450	0.072	405.190	< 0.001	4.261(3.700, 4.907)
**Education level (X3)**					
Illiterate					1.000
Primary	0.231	0.013	28.531	< 0.001	0.587(0.541, 0.653)
Junior high school and above	−0.353	0.045	62.350	< 0.001	0.702(0.643, 0.767)
**Marital status (X4)**					
Married					1.000
Not married	0.646	0.046	5.864	< 0.001	1.908(1.742, 2.090)
**Living at home**					
Yes					1.000
No	0.335	0.129	6.740	0.060	1.398(1.086, 1.801)
**Type of residence (X5)**					
Urban					1.000
Rural	0.490	0.046	115.169	< 0.001	1.632(1.492, 1.785)
**Caring for grandchildren (X6)**					
Yes					1.000
No	0.528	0.047	127.579	< 0.001	1.696(1.547, 1.859)
**Pain (X7)**					
Never					1.000
Occasionally	0.012	0.043	0.084	0.171	1.013(0.931, 1.101)
Often	0.331	0.052	40.742	< 0.001	1.392(1.257, 1.541)
**Live with children (X8)**					
Never					1.000
≤ 6 months	−0.064	0.072	0.793	0.089	0.938(0.814, 1.080)
>6months	0.255	0.044	33.110	< 0.001	1.291(1.183, 1.408)

### Decision tree analysis

**Figure 1:**
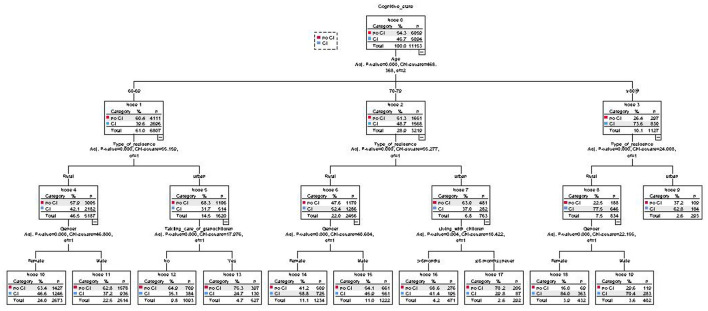
Decision tree analysis of the factors correlated cognitive impairment among older adults in China.

Those variables found to be significantly associated with CI in the logistic regression analyses were next used to conduct a decision tree analysis. CI was treated as the dependent variable (Y) in this model, while explanatory variables included gender (X1), age (X2), level of education (X3), marital status (X4), residence type (X5), participation in caring for grandchildren (X6), physical pain (X7), and living with children (X8). These decision tree analyses were conducted by adopting the CHAID pre-pruning method for tree pruning. The maximum tree depth of the model was set to 3, the minimum number of cases in the parent node was 100, and the minimum number of cases in the child was 50. The final decision tree (Figure 1) ultimately incorporated six explanatory variables including age (X2), type of residence (X5), gender (X1), participation in caring for grandchildren (X6), physical pain (X7), and living with children (X8), among which age (X2) had the most significant effect.

## Discussion

In this study, logistic regression analyses were used to identify factors associated with cognitive impairment among elderly individuals, revealing that gender, age, education level, marital status, residence type, living at home, physical pain, participation in taking care of grandchildren, and living together with children were the primary variables related to such impairment. Logistic regression analyses offer the advantage of allowing for the effective determination of the relevance of a given independent variable based on corresponding *P*-values, with OR values further allowing for the determination of the impact and directionality of independent variables on the dependent variable of interest. Decision tree analyses provide insight into the importance and influence of each included variable on the overall model, subdividing a given patient population based on a series of characteristics of interest. In the decision tree analysis in the present status, age, which had the greatest impact on cognitive impairment, served as the first layer of this model. Cognitive impairment rates were found to gradually rise with age, in line with prior data from other studies conducted in China and abroad (13, 36–38).

The second layer of the decision tree analysis in this study was place of residence, with cognitive impairment rates being higher in rural areas relative to urban areas. This aligns well-with prior studies reporting lower levels of cognitive impairment among elderly individuals in urban environments in China relative to those in rural environments (39–41).

The third layer of this decision tree analysis model consisted of gender, participation in taking care of grandchildren, and living with children. Cognitive impairment prevalence was higher among elderly women in rural areas relative to men at any age. This may be because women exhibit a longer life expectancy than men. In addition, cognitive impairment was established based upon a combination of MMSE scores and education level, and adults included in the 2018 CHARLS study that were over the age of 60 were thus born before 1960, corresponding to lower rates of educational attainment among women than among men. Whether cognitive impairment rates will decline with the continuing rise in female educational levels remains to be established and warrants further empirical investigation.

The variable ”taking care of grandchildren” was associated with significant differences in cognitive impairment among the elderly aged 60–69 in urban areas. Further analysis found a very interesting phenomenon: the incidence of cognitive impairment among those who did not take care of their grandchildren was 35.1%, which was significantly higher than that of those who took care of grandchildren (24.7%). One possible explanation may be that participating in caring for grandchildren produces bi-generational support such that these individuals may obtain positive emotional support and other reverse intergenerational support, consistent with the importance of such support in the maintenance of mental and physical health among elderly individuals Older adults not limited by disability or pain can also better participate in social activities, and both social participation and emotional comfort were protective factors associated with lower rates of cognitive impairment (42). However, these factors did not significantly impact cognitive impairment rates among elderly individuals in rural areas.

In urban elderly populations, adults over the age of 80 that lived with their children for > 6 months exhibited higher levels of cognitive impairment than observed for adults that had never lived with their children or had lived with their children for < 6 months. This may be the result of bias introduced by the fact that individuals exhibiting cognitive impairment were more likely to need to live with their children. Alternatively, daily care provided by these children may result in accelerated cognitive decline among older adults.

There were certain inherent limitations to the implementation of decision tree analyses. In particular, algorithm pruning can impact the accuracy with which unknown data were classified, with the difficulty of decision tree pruning rising as more factors were included in the corresponding analyses. In the present study, age was identified as the factor most closely associated with cognitive impairment in the elderly Chinese population, followed by place of residence (rural vs. urban) and gender, while caring for grandchildren and living with children were less robustly associated with this outcome variable. In China, income level was lower for individuals dwelling in rural areas relative to urban areas, and most of their children leave their hometown to work in the cities (43, 44), lack of emotional communication and financial support may affect the cognitive health status of rural elderly people lacking sufficient communication and emotional comfort. As such, health policymaking in China should focus on improving the cognitive health of elderly individuals in rural environments. While rates of cognitive impairment were found to be higher among women than men in rural areas, this same difference was not evident in urban environments, suggesting that preventative and intervention efforts in China should specifically target elderly women in rural areas.

There were other limitations in this study due to the non-independent data and cross-sectional data. Since the data analyzed in this article was based on CHARLS data (an open database), the method for selecting samples in the baseline survey was that if a household had persons older than 40, one of them was randomly selected. If the chosen person was 45 or older, then he/she became a main respondent and interviewed his or her spouse. So, the spouses were also included in the data set. If two people from the same household were both included in the data set, the data set would contain non-independent data. In addition, this study was a cross-sectional study, which only analyzed the factors correlating with cognitive impairment of the elderly and could not evaluate the causal relationship between the variables and cognitive impairment of the elderly. That was also one of the limitations of this study.

## Conclusion

The present study used significant variables derived from logistic regression analyses to facilitate the development of a robust decision tree analysis model capable of assessing cognitive impairment among elderly Chinese adults. The predicted risk score of the resultant decision tree was 0.237, which was smaller than that of the decision tree analysis with all possible variables included (0.389), indicating that logistic regression analyses enabled the selection of significant variables, optimizing the efficiency of classification and processing while minimizing associated predictive risk.

## Data availability statement

Publicly available datasets were analyzed in this study. This data can be found here: http://charls.pku.edu.cn.

## Ethics statement

The studies involving human participants were reviewed and approved by Ethical approval for all the CHARLS waves was granted from the Institutional Review Board at Peking University, China. The IRB approval number for the main household survey, including anthropometrics, is IRB00001052-11015. The patients/participants provided their written informed consent to participate in this study. Written informed consent was not obtained from the individual(s) for the publication of any potentially identifiable images or data included in this article.

## Author contributions

Y-yW was responsible for the conception, design of the study, quality control, review of the manuscript, and was responsible for the whole manuscript. X-xW was responsible for data collection and collation. MZ, SL, and HD were responsible for the manuscript writing and revision. All authors contributed to the article and approved the submitted version.

## Funding

This work was supported by grants from the Provincial Foundation for Excellent Young Talents of Colleges and Universities of Anhui Province (CN) (No. gxbjZD2020040).

## Conflict of interest

The authors declare that the research was conducted in the absence of any commercial or financial relationships that could be construed as a potential conflict of interest.

## Publisher's note

All claims expressed in this article are solely those of the authors and do not necessarily represent those of their affiliated organizations, or those of the publisher, the editors and the reviewers. Any product that may be evaluated in this article, or claim that may be made by its manufacturer, is not guaranteed or endorsed by the publisher.
